# Dermoscopic and Reflectance Confocal Microscopic Features of a Primary Cutaneous Anaplastic Large Cell Lymphoma (C-ALCL) of the Eyelid: A Case Report with Histopathologic Correlation

**DOI:** 10.3390/reports9020164

**Published:** 2026-05-21

**Authors:** Biagio Scotti, Cosimo Misciali, Martina D’Onghia, Alberto Gualandi, Sabina Vaccari, Federico Venturi, Elisabetta Magnaterra, Elisa Cinotti, Emi Dika

**Affiliations:** 1 Department of Medical and Surgical Sciences, Alma Mater Studiorum, University of Bologna, 40126 Bologna, Italy; 2 Oncologic Dermatology Unit, IRCCS Azienda Ospedaliero-Universitaria di Bologna, 40126 Bologna, Italy; 3 Dermatology Unit, Department of Medical, Surgical and Neurological Sciences, University of Siena, 53100 Siena, Italy

**Keywords:** primary cutaneous anaplastic large cell lymphoma, C-ALCL, ALCL, pcALCL, dermoscopy, dermatoscopy, reflectance confocal microscopy, RCM, imaging, histology, histopathology

## Abstract

**Background and Clinical Significance:** Primary cutaneous anaplastic large cell lymphoma (C-ALCL) is a CD30-positive T-cell lymphoproliferative disorder that can clinically resemble various non-melanoma skin cancers, making diagnosis challenging. Although histopathology remains the diagnostic gold standard, non-invasive imaging modalities such as dermoscopy and reflectance confocal microscopy (RCM) are increasingly used as complementary tools to support the differential diagnosis. To date, no data on RCM features of C-ALCL have been described. **Case Presentation:** We report the case of an 80-year-old man presenting with a rapidly enlarging nodule on the lateral aspect of his right eyelid, providing a detailed account of dermoscopic and RCM findings integrated with clinicopathological correlation. Dermoscopy revealed a red-orange homogeneous background with white streaks, and polymorphic vascular structures, while subsequent RCM (Vivascope 3000 probe) demonstrated marked architectural disarray of the epidermis and dermoepidemal junction, with prominent epidermal involvement characterized by aggregates of highly reflective cells. In the absence of alternative diagnostic patterns, these features raised suspicion for a cutaneous lymphoproliferative disorder, which was later confirmed by histopathological and immunohistochemical analyses. **Conclusions:** Our findings support the value of RCM as a practical tool in guiding differential diagnosis and biopsy, particularly for rapidly growing lesions located in anatomically sensitive areas.

## 1. Introduction and Clinical Significance

Primary cutaneous anaplastic large cell lymphoma (C-ALCL) is a CD30-positive T-cell lymphoproliferative disorder (LPD) of the skin that generally carries a favorable prognosis in the absence of systemic involvement [1]. CD30-positive LPDs account for approximately 25–30% of all primary cutaneous lymphomas, representing the second most common clonal T-cell neoplasm of the skin after mycosis fungoides (MF) [2].

From a clinicopathological perspective, these entities are considered part of a spectrum including C-ALCL and lymphomatoid papulosis (LyP), which share overlapping histopathological features but significantly differ in clinical behavior [2]. The differential diagnosis also includes other cutaneous T-cell lymphomas, such as MF with large-cell transformation, as well as secondary cutaneous involvement by systemic large-cell lymphomas [1,2,3]. Therefore, accurate diagnosis requires integration of clinical, histopathological, immunophenotypic, and disease course data, making C-ALCL particularly challenging to diagnose in routine practice [3].

Although histopathological examination remains the gold standard for the diagnosis of primary cutaneous lymphomas, there is growing interest in the use of non-invasive diagnostic imaging techniques, including dermoscopy and reflectance confocal microscopy (RCM) [4]. Rather than providing a cytological or lineage-specific diagnosis, RCM enables in vivo assessment of architectural patterns and cellular features, supporting clinical suspicion and guiding biopsy to the most representative areas (i.e., those with higher tumor cell density), particularly in anatomically delicate sites [5].

To date, the literature on C-ALCL remains limited, with a PubMed and Scopus search identifying only a few primary studies describing its dermoscopic features [6,7,8,9], while the diagnostic utility of RCM has yet to be defined. To date, no data on RCM features of C-ALCL have been reported. In this context, we aimed to assess the contribution of dermoscopy and, particularly, RCM, with special emphasis on its correlation with conventional histopathology in eyelid C-ALCL.

## 2. Case Presentation

**Figure 1:**
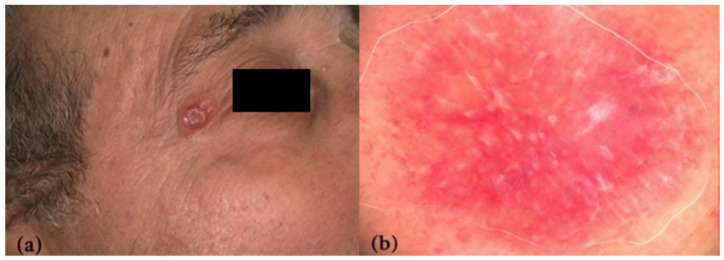
(**a**) Clinical image of a skin-colored to erythematous nodule lateral to the right outer canthus. (**b**) Red-orange homogeneous background with peripheral fading, whitish streaks, and polymorphic vessels at dermoscopy (20× magnification).

An 80-year-old man presented to our clinic with a reddish, painless nodule on the lateral aspect of his right upper eyelid, which had progressively developed over the past three months. On clinical examination, an 18 × 12 mm skin-colored to erythematous nodule with fine scaling was observed lateral to the outer canthus of the right eye; the rest of the surrounding skin appeared unremarkable (Figure 1a).

Contact, polarized dermoscopy (20× magnification, VivaCam D200, VivaScope GmbH, Munich, Germany) showed a red-orange homogeneous background with shiny white structures (predominantly streaks), along with polymorphic vessels, more prominent at the periphery (Figure 1b).

Reflectance confocal microscopy (RCM) was performed in the central portion of the lesion using the VivaScope 3000 (MAVIG GmbH, Munich, Germany; Lucid-Tech Inc., Henrietta, NY, USA), a near-infrared laser system with a wavelength of 830 nm; vertical stacks of 750 × 750 µm were acquired.

**Figure 2:**
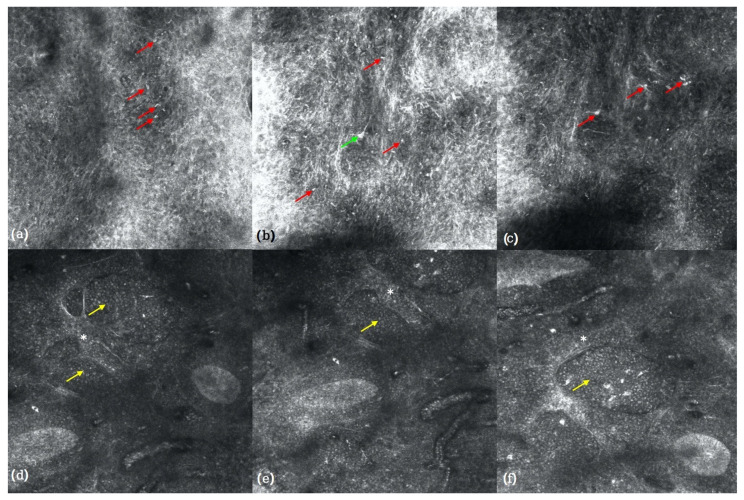
RCM images (350× magnification) showing large, highly reflective cells with areas of prominent involvement within the epidermis and along the dermoepidemal junction (red arrows), associated with disruption of the normal epidermal architecture and loss of the regular honeycomb pattern (**a**–**c**). Scattered dendritic-shaped hyperreflective structures are also visible (green arrow). At the superficial dermal level, compact aggregates of medium-sized, monomorphic, moderately to highly reflective cells (yellow arrows) are observed, embedded within a fibrous stromal background characterized by thickened, highly reflective bundles (white asterisks) (**d**–**f**).

The examination revealed large, highly reflective cells with areas of prominent epidermal involvement within the dermoepidemal junction (red arrows), leading to a disruption of the normal epidermal architecture and loss of the regular honeycomb pattern. In addition, scattered dendritic-shaped hyperreflective structures were observed (green arrow) (Figure 2a–c). At the superficial dermal level, compact aggregates of medium-sized, monomorphic, moderately to highly reflective cells were detected (yellow arrows), embedded within a fibrous stroma characterized by bright, thickened bundles, consistent with altered stromal architecture (white asterisks) (Figure 2d–f).

**Figure 3:**
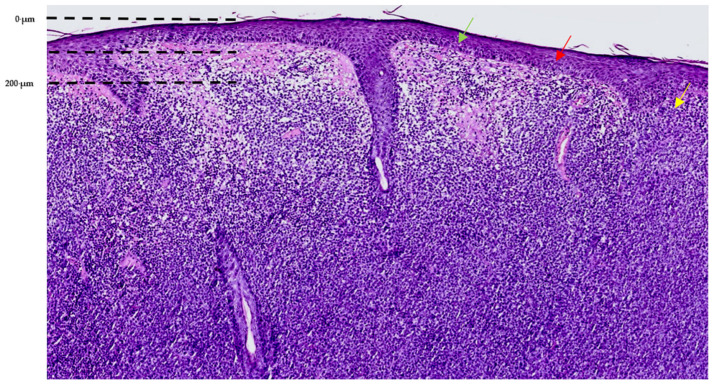
(H&E stain, 7×) Histopathological examination showing a diffuse dermal lymphoid proliferation involving the full thickness of the dermis, without sharp circumscription and partial distortion of the overlying epidermal architecture. Color-coded arrows indicate the correspondence with RCM findings, highlighting cellular elements at both the epidermal and dermal levels.

**Figure 4:**
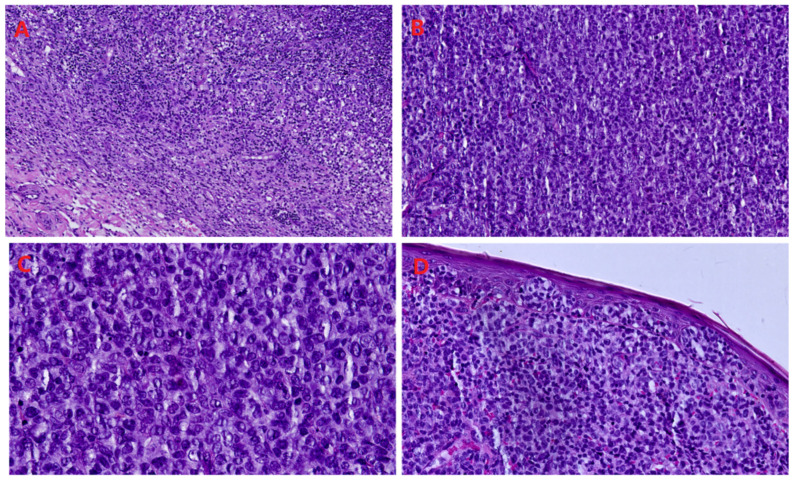
Histopathological features (H&E stain). (**A**) Diffuse dermal lymphoid infiltrate with a sheet-like growth pattern (10× magnification); (**B**) dense, relatively monomorphic cellular proliferation (20× magnification); (**C**) large atypical lymphoid cells with marked nuclear pleomorphism and prominent nucleoli (40× magnification); (**D**) epidermal involvement with focal intraepithelial lymphocytosis and partial architectural distortion (20× magnification).

After surgical excision, the histopathology displayed a diffuse dermal lymphoid infiltrate (Figure 3) with a sheet-like growth pattern, composed of a dense proliferation of large atypical lymphoid cells with marked cytological atypia, including irregular nuclear contours, prominent nucleoli, and high mitotic activity (Figure 4). The cellular population appeared relatively monomorphic at lower magnification but displayed marked nuclear pleomorphism at higher magnification. Epidermal involvement was present, with focal intraepithelial lymphocytosis and partial distortion of the epidermal architecture; the papillary dermis showed stromal changes, including edema and thickened collagen bundles, associated with prominent superficial blood vessels (Figure 3 and Figure 4).

**Figure 5:**
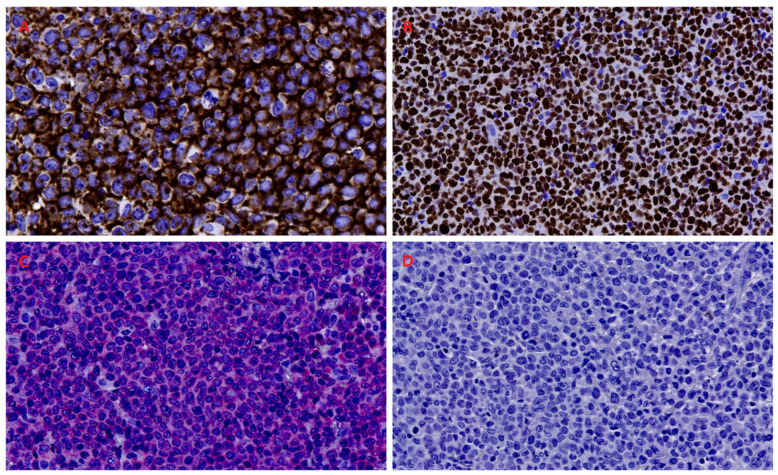
Immunohistochemical characterization. (**A**) Dense dermal proliferation of atypical lymphoid cells showing strong, diffuse CD30 expression, highlighting the neoplastic component (40× magnification); (**B**) Ki-67 staining demonstrates a markedly elevated proliferative index (20× magnification); (**C**) diffuse CD3 positivity consistent with a T-cell phenotype (20× magnification); (**D**) absence of ALK expression (20× magnification).

Immunohistochemically, the neoplastic cells showed strong and diffuse CD30 expression, highlighting the tumor component, and were positive for CD43 with aberrant CD3 expression, consistent with a T-cell phenotype. They were negative for ALK, CD4, and CD8. The proliferative index (Ki-67/MIB-1) was markedly elevated, involving more than 75% of neoplastic cells (Figure 5). Overall, after excluding systemic involvement by comprehensive whole-body imaging, these findings were consistent with a diagnosis within the spectrum of primary cutaneous CD30-positive lymphoproliferative disorders, with features supporting C-ALCL.

## 3. Discussion

To the best of our knowledge, this is the first study to characterize both the dermoscopic and RCM features of C-ALCL and to relate them to the corresponding histopathologic findings. Previous reports have described the dermoscopic patterns of C-ALCL, typically showing pink-to-yellow/orange structureless areas or a salmon-colored background with polymorphic or linear irregular vessels, shiny white structures, ulceration, and hemorrhagic areas [6,7,8,9].

Consistent with our findings, dermoscopy may support the recognition of a malignant non-melanocytic lesion; however, the corresponding RCM characteristics in this setting remain largely unexplored.

RCM is a noninvasive imaging technique with cellular resolution (lateral resolution 0.5–1 µm; optical sectioning 3.5–4 µm) that enables in vivo visualization of horizontal skin sections, with a maximum penetration depth of 200 μm [10]. It is approved for dermatologic use in both the United States and Europe, having received regulatory clearance from the FDA and the EMA [11,12].

Although RCM’s primary application concerns the diagnosis of melanocytic lesions, its role has also been investigated in the diagnosis and treatment monitoring of cutaneous lymphomas, mainly cutaneous T-cell lymphomas (CTCL), as B-cell lymphomas are largely confined to the dermis and therefore less amenable to RCM evaluation [5].

Interestingly, several studies have focused on characterizing the RCM features of MF, the most common form of CTCL [13,14,15,16,17,18,19], providing detailed descriptions of both epidermal and dermal alterations and highlighting the potential of RCM to support early diagnosis, assess disease activity, and monitor therapeutic response. Specifically, Koller et al. assessed the diagnostic accuracy of RCM in erythematous–squamous skin diseases, reporting a specificity of 92.9% for MF [14]; whereas Melhoranse Gouveia et al. demonstrated that a combination of four RCM features (Pautrier’s microabscesses, epidermal and junctional lymphocytes, and interface dermatitis) constituted an RCM checklist for MF that predicted disease severity with excellent accuracy, corresponding to an AUC of 0.95 (*p* = 0.003) [19]. In addition, the same group reported a case of transformed MF assessed by in vivo RCM, demonstrating the tools’ ability to identify transformed atypical lymphocytes as large, bright, round cells [17,18]. Finally, Yeager et al. described epidermal disarray in tumor-stage MF, characterized by weakly refractile round-to-oval cells within the spinous layer and at the dermoepidemal junction [16].

In our case of C-ALCL, RCM revealed hyper-reflective roundish cells and large cellular aggregates within the epidermis (corresponding histopathologically to focal aspects of intraepithelial lymphocytosis) and at the dermoepidemal junction, as well as dense hyper-reflective aggregates within the superficial dermis, consistent with a dense lymphoid proliferation on histopathology. The distinction between the hyperrefractive lymphocytes and other potential hyperreflective cells on RCM may be suggested based on morphological features and cellular distribution: melanophages typically display a triangular (plump) morphology rather than a round shape, whereas dendritic cells are pleomorphic and tend to be scattered [20,21]. Moreover, keratin debris, although highly reflective on RCM, lacks an organized cellular structure (appearing amorphous) and is typically confined to the stratum corneum or epidermal invaginations [22].

These observations are consistent with previous reports indicating that larger lymphocytes are more readily detectable by RCM, supporting the potential value of this technique in challenging diagnostic settings. Accordingly, particularly when integrated with dermoscopy and clinical context, RCM may assist in improving diagnostic accuracy. In this setting, the selection of immunohistochemical markers was guided by their diagnostic relevance, and only the most contributory findings were reported, in line with routine dermatopathological practice.

RCM may aid in distinguishing C-ALCL from other malignancies that can mimic it clinically and dermoscopically, such as Merkel cell carcinoma (MCC) [23]. Although MCC may exhibit shiny white structures on dermoscopy and increased fibrotic stroma on RCM, its tumor cells typically appear smaller and hyporeflective [23]. More broadly, the differential diagnosis of malignant periocular tumors includes amelanotic melanoma (AM), squamous cell carcinoma (SCC), basal cell carcinoma (BCC), and sebaceous carcinoma (SC) [24,25,26,27,28,29].

On RCM, AM is characterized by widespread pagetoid infiltration of roundish atypical cells within the epidermis and non-edged dermal papillae at the dermoepidemal junction [21,25]. SCC shows disruption of epidermal architecture with keratinocyte detachment, dyskeratotic cells, and increased vascularity [22,30]. BCC typically presents as aggregates of refractile tumor cells with elongated, monomorphic basaloid nuclei arranged in a palisading pattern, disrupting the normal honeycomb architecture of the epidermis and superficial dermis [31]. To date, no specific RCM features have been described for SC.

**Figure 6:**
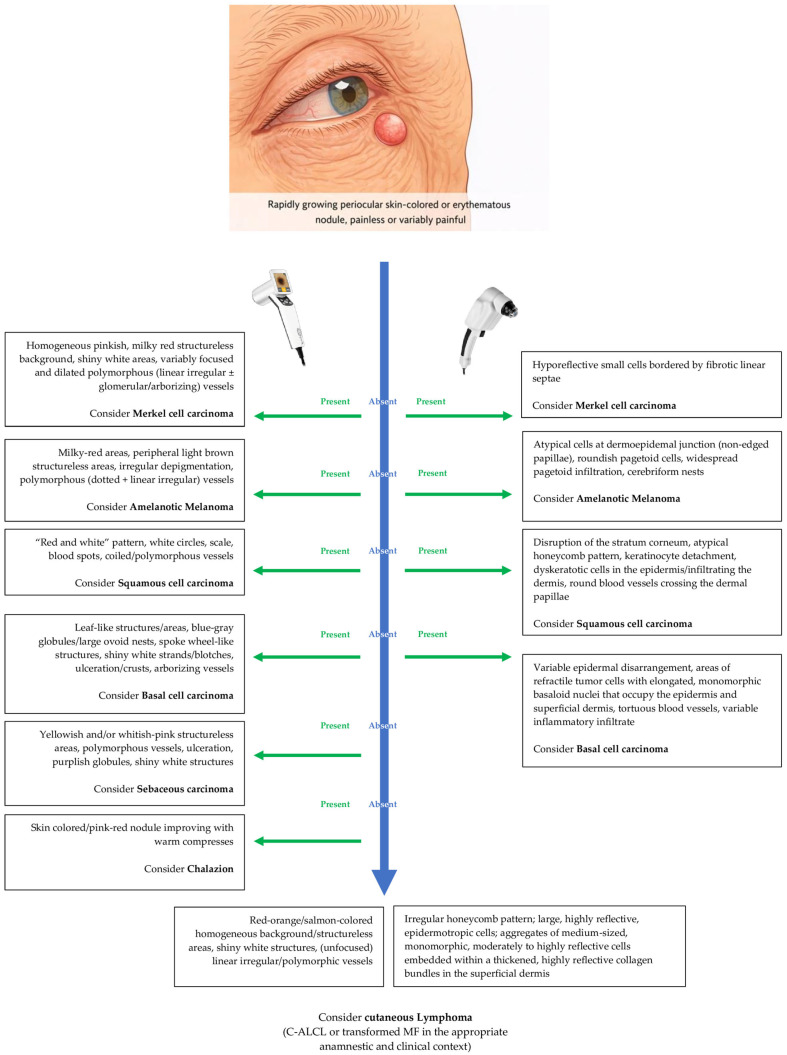
Practical dermoscopy–RCM flowchart for suspected cutaneous lymphomas.

From a clinicopathological standpoint, C-ALCL may also mimic benign or reactive conditions, such as keratoacanthoma-like lesions and inflammatory nodules (i.e., inflamed chalazion) [32], complicating the initial diagnosis. It may present with pseudocarcinomatous hyperplasia or a prominent inflammatory background, leading to confusion with squamous neoplasms or reactive processes [33,34], or can arise in the setting of chronic inflammatory conditions (e.g., erythema nodosum), further obscuring the underlying lymphoid neoplasm [35]. Moreover, the differential diagnosis of the CD30-positive cutaneous LPDs includes arthropod bite reactions and cutaneous pseudolymphomas; however, these conditions typically exhibit a self-limited course and a polymorphous infiltrate, in contrast to the persistent and nodular proliferation of large atypical lymphoid cells seen in C-ALCL [36]. In Table 1, we summarized the major differential diagnoses of periocular skin-colored/erythematous tumors, while in Figure 6, we proposed a practical flowchart integrating dermoscopic and RCM features for the evaluation of suspected cutaneous lymphomas.

Several limitations should be acknowledged. In addition to those inherent to the single-case design and the lack of blinded multi-reader RCM assessment, the penetration depth of RCM is intrinsically limited, particularly in the presence of dense dermal infiltrates that may extend beyond the imaging range, representing an important caveat in nodular lesions with negative findings. Furthermore, hyperreflective cells may be subject to misclassification, as similar features can be observed in non-neoplastic elements. Finally, epidermal alterations (e.g., hyperplasia, spongiosis, or scaling) may further impair visualization of deeper dermal structures.

## 4. Conclusions

RCM may help identify distinct architectural patterns and hyper-reflective cellular aggregates in C-ALCL, potentially assisting clinicians as an adjunctive tool not only in diagnosis but also in disease monitoring. By providing real-time, in vivo information on epidermal and superficial dermal architecture, RCM may support clinical suspicion and help guide biopsy toward the most representative areas. However, further studies on larger cohorts are warranted to better characterize the diagnostic accuracy, specificity, and overall clinical utility of RCM in this specific clinicopathological setting.

## Figures and Tables

**Table 1:** Noninvasive imaging features and immunohistochemical profiles of the most common periocular primary tumors.

Diagnosis	Dermoscopic Features	RCM Hallmarks	IHC	References
C-ALCL	Red-orange homogeneous background or structureless areas; shiny white structures; (unfocused) linear, irregular/polymorphic vessels.	Loss of the regular honeycomb pattern; large, highly reflective cells with areas of prominent epidermal involvement within the epidermis and along the dermo-epidermal junction; aggregates of medium-sized, relatively monomorphic, moderately to highly reflective cells embedded within thickened, highly reflective collagen bundles in the superficial dermis	CD30 (>75%), variable T cell phenotype (CD3/CD4/CD8), ALK (−);	[6,7,8,9]
Merkel cell carcinoma	Homogeneous pinkish, milky-red structureless background; shiny/not-shiny white areas; variably focused and dilated polymorphous (linear irregular ± glomerular/arborizing vessels).	Hyporeflective small cells bordered by fibrotic linear septae.	CK20 (+, paranuclear dot), TTF-1 (−), synaptophysin, INSM1, chromogranin; MCPyV T-antigen.	[23]
Amelanotic melanoma	Milky-red areas; peripheral light brown structureless areas; irregular/subtle depigmentation; polymorphous (dotted + linear irregular) vessels.	Atypical cells at dermoepidemal junction (non-edged papillae); roundish pagetoid cells; widespread pagetoid infiltration; cerebriform nests.	S100 (+), Melan-A (variable), HMB-45 (variable), SOX10 (+), PRAME (+).	[21,24,25,26]
SCC/Keratoacanthoma (KA)	Keratin (central in KA); white circles (highest specificity); blood spots; scale; “red and white” pattern; coiled vessels; polymorphous vessels (SCC); radial hairpin vessels (KA).	Disruption of the stratum corneum with evident parakeratosis; alteration of the epidermal pattern with severe keratinocyte detachment; round, nucleated dyskeratotic cells in the epidermis and occasionally infiltrating the dermis; round blood vessels crossing the dermal papillae.	p40 (+), p63 (+), CK5/6 (+).	[27,30]
BCC	Leaf-like structures/areas; multiple blue-gray globules (not aggregated); large blue-gray ovoid nests; scattered dots; spoke wheel-like structures; shiny white strands/blotches; ulceration; arborizing vessels.	Variable epidermal disarrangement (loss of the honeycomb pattern and nuclear pleomorphism of the keratinocytes), areas of refractile tumor cells with elongated, monomorphic basaloid nuclei with nuclear streaming occupying the epidermis and superficial dermis; increased dermal vasculature with dilated and tortuous blood vessels; variable inflammatory infiltrate associated with the tumor.	BerEP4 (+), BCL2 (+).	[28,31]
Sebaceous carcinoma	Yellowish structures; yellowish structureless areas; whitish-pink areas; polymorphous vessels; ulceration; purplish globules; shiny white structures.	None yet reported.	Adipophilin (100% sensitive), EMA (+), CK7 (+), Ber-EP4 (−/weak), AR	[29]
Chalazion	Skin colored/pink-red nodule, variably painful	None yet reported.	Generally not requested	[32]

## Data Availability

The data that support the findings of this study are available from the corresponding author upon reasonable request due to privacy concerns.

## Abbreviations

The following abbreviations are used in this manuscript:

ALK	Anaplastic lymphoma kinase
AM	Amelanotic melanoma
AR	Androgen receptor
AUC	Area under the curve
BCC	Basal cell carcinoma
BCL2	B-cell lymphoma 2
BerEP4	Epithelial cell adhesion molecule (EpCAM) antibody
C-ALCL	Primary cutaneous anaplastic large cell lymphoma
CD	Cluster of differentiation
CK	Cytokeratin
CTCL	Cutaneous T cell lymphomas
EMA	European Medicines Agency
FDA	Food and Drug Administration
HMB45	Human melanoma black 45
H&E	Hematoxylin and eosin
INSM1	Insulinoma-associated protein 1
LPD	Lymphoproliferative disorder
LyP	Lymphomatoid papulosis
MCC	Merkel cell carcinoma
MCPyV	Merkel cell polyomavirus
MIB-1	Antibody against Ki-67 antigen
MFs	Mycosis fungoides
RCM	Reflectance confocal microscopy
SC	Sebaceous carcinoma
SCC	Squamous cell carcinoma
SOX-10	SRY (Sex-determining Region Y)-box 10
TCR	T-cell receptor
TTF-1	Thyroid transcription factor-1
